# Detection of High Abilities: An Empirically Evidenced Alternative to Biased Detection

**DOI:** 10.3390/jintelligence14010009

**Published:** 2026-01-06

**Authors:** Leire Aperribai, Elena Rodríguez-Naveiras, Triana Aguirre, Teresa González-Pérez, África Borges

**Affiliations:** 1 Department of Clinical and Health Psychology and Research Methodology, University of the Basque Country UPV/EHU, 20018 Donostia-San Sebastián, Spain; 2 Department of Clinical Psychology, Psychobiology and Methodology, University of La Laguna, 38200 San Cristóbal de La Laguna, Spain; naveiras@ull.edu.es (E.R.-N.); taguirre@ull.edu.es (T.A.); aborges@ull.edu.es (Á.B.); 3 University Institute for Women’s Studies, Department of History and Philosophy of Science, Education, and Language, University of La Laguna, 38200 San Cristóbal de La Laguna, Spain; teregonz@ull.edu.es

**Keywords:** detection, gender, high ability, identification, instruments, students

## Abstract

Students with high ability (HA), due to their differential characteristics, need to receive a specific educational response for the adequate development of their potential. Thus, they must be detected and then identified, but many of these students (around 9.5%, based on prevalences of domain-specific definitions) remain unidentified, especially among girls. The low detection of highly able students raises the need to establish more objective and efficient criteria. Thus, the objective of this study is to analyze whether the use of objective tests in the procedure increases the number of male and female students detected with HA. To detect students with HA, the general intelligence assessment instrument Matrices-TAI has been applied to students from the first to the third year of Compulsory Secondary Education in different educational centers in the Community of the Canary Islands (N = 1216). The results show that in official data, only 1.17% of HA students (0.89% of girls and 1.44% of boys) have been identified, while 9.21% (8.10% of girls and 10.35% of boys) have a higher intelligence in this convenience sample, coinciding with the percentages of talent found in the literature. In conclusion, in our sample, universal screening with a rigorous intelligence test identified a substantially larger proportion of students, including girls, than current nomination-based procedures appear to capture in administrative statistics, suggesting that such screening may reduce gender disparities in identification.

## 1. Introduction

The specialized literature points out that students with high abilities (HA), due to their differential characteristics, need a specific educational response to foster the complete development of their potentialities (Comes et al., 2008; Martin-Lobo et al., 2018; Rodríguez-Naveiras et al., 2019; Sastre-Riba, 2012; Vaivre-Douret, 2011). This is provided by the Organic Law of Education (LOE, 2006), which categorizes these students within those with specific needs. For these students, detection and identification are essential to receive the educational response they need. Likewise, it is especially important for this to be performed from an early age (Elices et al., 2006; LOE, 2006). An adequate educational response helps to improve academic performance and prevents school failure in these students (A. M. Rodríguez, 2013; Valdés et al., 2013).

The definition of the concept of high ability has been given by many theoretical models, but it has not reached a consensus among the scientific community (Sastre-Riba, 2008). As an example, Covarrubias and Marín (2015) conceptualize a highly able student as a scholar with a high and multidimensional intellectual potential, while the Government of the Canary Islands considers that a student over 12 years old with high ability, has a high level of cognitive skills in the following fields: logical reasoning, perceptual management, memory management, mathematical reasoning and spatial aptitude (Consejería de Educación, Ciencia y Universidades, n.d.).

The diversity of theoretical models and definitions has hindered the existence of a standardized response for the identification of this student body. In the case of Spain, each Autonomous Community has its own legislation on high ability (Hernández & Gutiérrez, 2014; Quílez & Lozano, 2020). However, it is quite common that the final assessment is performed in two phases: the first, detection, followed by the second phase, called identification (Valadez et al., 2020). The detection is performed by the relevant people in the context of the student: teachers, fathers and mothers, who are responsible for pointing out the possible presence of a high ability in students (Rubenstein & Ridgley, 2017; Zaia et al., 2018). This process can be carried out in a non-formalized way, simply informing of the possible capacity, or it can be formalized through the completion of questionnaires, both by parents and teachers (Sánchez & Baena, 2017). The identification, which is the next step, seeks confirmation of high ability in the student (Johnsen, 2009) who has been previously detected. This last process is carried out by the psychological and educational orientation team of the educational center through objective tests (Belur & Oğuz-Duran, 2017; Covarrubias & Marín, 2015), some of the most used being the Wechsler Intelligence Scale (Wechsler, 2015), the Raven Progressive Matrices Test (Raven et al., 1993), the Catell g factor test (Cattell & Cattell, 1994) or the Differential and General Aptitudes Battery (Yuste et al., 2002).

There is a discrepancy about the prevalence of high ability and talents in the population, but many authors have considered prevalences based on domain-specific explanations, such as Gagné’s (1985) definition, understanding giftedness as a potential that is expressed in at least one domain, and talent as a superior mastery of skills expressed in at least one area of human activity. Based on these kinds of conceptual frameworks, it is estimated that this prevalence is around 10% (Hernández & Gutiérrez, 2014). Despite the importance given by the experts to early identification, which is also reflected in the Spanish legislation for over 20 years (Hernández & Gutiérrez, 2014; Quílez & Lozano, 2020), in the course 2020–2021 (see Table 1), taking into account the total number of non-university students (8,232,295), only 40,916 students have been identified in Spain, which is 0.49%. In relation to the data from the Canary Islands, only 2393 out of the total number of registered students (340,576) have been identified, which accounts for only 0.70%.

There are several reasons for this low prevalence of identification. The lack of consensus previously mentioned regarding the definition of the concept can be one of them. It must also be admitted that the concept of high ability is biased by the existence of stereotypes and myths about high abilities that further hinder the conceptualization of HA and, consequently, their detection (Pérez et al., 2020). Although the myths are multiple and varied, among them are those that refer to the characteristics of this student body as girls and boys rejected by their peers and with communication and relationship difficulties, or as people with a great capacity to lead groups. Pérez et al. (2020) stress that these are myths that are difficult to change, rooted in social representations. It is important to understand that the teaching staff is not free from these representations, and that when they detect them, they will tend to look for these biased characteristics and neglect those that could better describe the students with HA. Teachers themselves admit the persistence of the existence of myths and stereotypes in society, as well as the difficulties in detection or nomination, and the lack of training in this regard (Aperribai & Garamendi, 2020). Therefore, a biased detection process, based on teachers’ and parents’ perceptions, may lead to an underdetection of HA students, and consequently to an underidentification of them (Card & Giuliano, 2016).

This situation is questionable, as one should wonder what happens to this 9.5% of students who remain unidentified in Spain, but the problem is aggravated when the prevalence is differentiated by gender (see Table 1). Among the identified students in Spain (0.49%), the majority refer to the male gender (0.32%), being the female identification still lower (0.17%). 

Taking into account that identification is partly based on intelligence tests, and that the level of intelligence does not differ between genders (Dolan et al., 2006; Van der Sluis et al., 2006, 2007), this lower identification of women in the number of students evaluated has no explanation, so it is necessary to question the reason for this disparity, which should be sought in the socialization of gender along with the scarce training of teachers that influences detection and, consequently, identification (Muñoz, 2018). The educational centers detect and propose a higher number of male students than of female students for the assessment of high abilities, which is why there is a deficient assessment or identification in female students, with very low rates (Muñoz, 2018). 

According to Bian et al. (2017), women perceive themselves and other women as less intelligent than men. Gender bias is conditioned by the internalization of roles and gender stereotypes. Gender stereotypes are the set of preconceived ideas used to explain the behavior of men and women, generated around the idea of how they should behave, and the roles they should play in the work, family, school, in public space, as well as how they should relate to each other (Lameiras et al., 2013). Gender stereotypes are inculcated unconsciously from birth, determining the behavior of boys and girls according to their sex. Thus, boys and girls, from the early birth, are projected onto the social roles of men and women which are characteristic in the culture in which they have been birth (Álvarez et al., 2017). The school, as an institution, reproduces the sexism that is present in society, so that its presence is detected in the teaching practice, in the attitudes of the faculty, and in the academic curriculum. The school continues to maintain beliefs and representations that do not question or propose alternative models to traditional (sexist) visions regarding the construction of identity and the assignment of male and female roles (Álvarez et al., 2017).

In several studies carried out on teachers, it has been confirmed that different behaviors regarding gender and gender stereotypes in educational settings are maintained (Siegle et al., 2010). Stereotypes are transmitted from the hidden curriculum through the way of acting in the center and/or the classroom, so that men are valued for their accomplishments, while women are further strengthened in their behavior. Teachers have a differentiated treatment for girls, and they receive less attention compared to boys (Fernández et al., 2011). The primacy of the male remains present in the educational centers; sexism and power relations in the classrooms have not been overcome. Not even the effect of positive actions and the promotion of regulations for equal opportunities has achieved co-education, so that social evolution does not involve an educational parallelism (Aguilar-Ródenas, 2013; Subirats, 2014).

As already mentioned, detection is the step before identification or assessment; it is carried out by the teachers and parents of students (Valadez et al., 2020), selecting those students who, according to the detection process (formal or informal), could have HA. However, the literature indicates that the teaching staff is not very precise when detecting these students (Valadez-Sierra et al., 2017), because both HA stereotypes (Pérez et al., 2020; Valadez-Sierra et al., 2017) as well as gender hinder the detection of students (Hyde, 2014). Although there is a tendency to deny that even today teachers maintain gender bias, there is a difference in the treatment offered to students, in the allocation of tasks according to gender, and in the promotion of competitiveness in boys, while passivity in girls (Ayala & Mateo, 2005). Regarding their capabilities, Rocha et al. (2010) indicate that teachers have different expectations according to gender, as they believe that women should compensate for their intellectual difficulties by working harder. In addition, there is a lack of confidence in their ability to manage ICT (Díaz de Greñu & Anguita, 2017). But also, fathers and mothers have expectations regarding their sons and daughters influenced by gender stereotypes (Rocha et al., 2010). 

Therefore, and as mentioned previously, the detection process leading to the assessment or identification is not entirely effective, as most students with HA (around 9.5%, based on domain-specific definitions) are not identified (Hernández & Gutiérrez, 2014), with the consequent lack of attention to their needs and the consequences that this entails (Araque & Barrio, 2010; Card & Giuliano, 2016). This reality questions the effectiveness of detection, establishing a filter that marks the previous selection through people who are not always trained to determine the presence of high abilities. Therefore, the need to establish more objective and efficient criteria is raised. 

At the international level, there are many experiences in identification. On the one hand, selection (detection) processes like those used in Spain have been used, but they need a better understanding of the relationships between selection (detection) procedures and the final results of the decision (identification) based on intelligence tests (McIntosh et al., 2018), as it happens in our context. On the other hand, in those educational systems that use a single composite score of a cognitive test as the main criterion to determine high ability, the experiences are diverse and controversial, based on the theories and instruments employed. Due to advances in psychometrics, one of the theories of intelligence that has been refined is the Cattell-Horn Carroll (CHC) model (Schneider & McGrew, 2012). The CHC theory is based on a hierarchical model of three strata: the third, which would be found at the top, would correspond to the factor g or general capacity; the second, would correspond to a broad stratum, where different intellectual skills would be collected (i.e., fluid intelligence factors); the third, would correspond to a specific stratum, where more specific factors would be collected (i.e., inductive reasoning) (Abad et al., 2020). However, according to McIntosh et al. (2018), even when its use at the international level is expanding, in Spain it is limited, and has not been previously used for direct detection or the selection phase.

Therefore, this study aims to analyze the extent to which the percentage of HA detection increases when the detection is made by measuring intelligence directly using the Cattell-Horn Carroll (CHC) model in the Autonomous Community of the Canary Islands. Thus, the objective of the study was to determine if there is a difference in the number of students detected with HA when it is performed through objective tests in our convenience sample, compared to the official prevalences of identified HA students (previously detected by following the formal detection processes before mentioned).

## 2. Materials and Methods

### 2.1. Methods and Design

A double methodology has been used to determine if there is a difference in the number of students detected with HA when performed through objective tests. On the one hand, documentary methodology has been used to extract information from census data provided by the Ministry of Education, Vocational Training and Sports (Ministerio de Educación, Formación Profesional y Deportes, n.d.), for both, the total of detected students in Spain and in the Autonomous Community of the Canary Islands. On the other hand, a cross-sectional survey methodology has been used for the collection of socio-demographic and intelligence data.

### 2.2. Participants

The sample was obtained thanks to the participation of 14 educational centers (10 public and 4 private) in the Canary Islands (Tenerife, Gran Canaria, La Palma and Lanzarote). The sample was selected by convenience, and a total of 1235 students from the first to third year of Compulsory Secondary Education (ESO) participated, whose ages range from 12 to 16 years (see Table 2).

It is worth mentioning that 19 of the participants (IQ *M* = 100.29; *SD* = 14.63) did not identify their gender as male or female (see Table 2). Since the study is based on the comparison of identification based on the female or male gender, the data of this student body was not taken into account for the analysis. Therefore, the final sample consisted of 1216 students.

### 2.3. Instrument

The Matrices-TAI test was used in this study. This General Intelligence Adaptive Test (Abad et al., 2020), is a computerized adaptive test that measures general intelligence (g factor). It provides the intellectual quotient or IQ of the participants, with confidence intervals, as a measure of general intelligence based on the assessment of capabilities to solve complex and new problems related to fluid intelligence (factor Gf) by resolving graphic matrices. The instrument has been created based on the Cattell-Horn-Carroll theoretical paradigm and the Item Response Theory psychometric paradigm. This version of the test has a pool of 149 items and its corresponding estimated parameters (a, b and c), and is performed online, so that the questions are adapted to the responses of the individuals. In its development, items’ difficulty (P) and discrimination (D) statistics were estimated to include only those items with adequate parameters. Also, unidimensionality was analyzed by carrying out a confirmatory factor analysis, where adequate fit indices were obtained (Abad et al., 2020). Furthermore, the instrument has obtained good indices of internal consistency (above 0.85 reliability indices) and good test–retest reliability estimates (an average of 0.82) in its paper version. It has been validated and standardized in a representative sample (N = 12,211) of the Spanish population (age range = 6 to 74 years). In this study, this instrument has been used for the detection of HA, taking as a cutoff point the IQ of 120, so it has been considered that participants with an IQ of 120 or higher can be detected as being highly able (HA) students, and students who do not reach the cutoff point do not meet this condition. In some educational and research contexts, an IQ of 130 or greater has been considered as a cutoff point to consider the high ability in a student. Nevertheless, in this study, the threshold of an IQ of 120 or higher has been considered for many reasons: on the one hand, the aim of this study is to analyze whether the objective test Matrices-TAI could serve as a universal screening instrument to detect HA. The IQ of 130 corresponds to a percentile of 98–99 (very high category) which would be too restrictive to detect the estimated 10% of the gifted and talented in the population (based on the domain-specific definition), while the IQs of 115–129 correspond to a percentile of 84–97 (high category), which better corresponds to the proportion of HA in the population. On the other hand, the main aim of detection and identification is to respond to the specific educational needs of HA students. As Ruf (2005) explains, an IQ of 120 would be in a first level of moderate potential that should be given an educational response, such as curricular enrichment. Therefore, detection and identification of students at this first level should be necessary for educational purposes. Thus, in this study, students with an IQ of 120 or superior have been taken into account to be considered as HA students, in Ruf’s all potential levels. 

### 2.4. Procedure

Firstly, the authorization of the corresponding university’s ethical committee for human research, the so-called Animal Research and Welfare Ethics Committee (CEIBA 2021-0449) was requested. Once the favorable report was obtained, the sampling process began by contacting the educational centers in the Autonomous Community of the Canary Islands. A total of 23 centers were contacted and 14 of these agreed to participate. Then, and in accordance with Article 7.1. of the Organic Law 3/2018, of December 5, on the protection of Personal Data and Guarantee of Digital Rights, parents or legal guardians of students up to 13 years old were asked to give informed consent, while, as established by the regulation, students over 14 years old were able to give their consent to participate in the research. 

After the consents were obtained, the researchers’ team, who were previously properly trained, accessed the classrooms of the participating educational centers to perform the collective test applications during regular class time. Once the researchers explained the research objectives to the students, they prepared the electronic devices for the test application and proceeded to supervision and data collection.

The participants were divided into groups of between 10 and 30 students, to whom the Matrices-TAI test was applied online on computers or tablets, with a maximum duration of 30 min. The presence and supervision of the team was of vital importance because it provided technical support to the participants in the face of the difficulties they encountered during the test application and to those participants with disabilities.

### 2.5. Data Analyses

On the one hand, we analyzed the frequencies and percentages of the students officially identified with HA, in relation to the total of the students of the first three courses of Compulsory Secondary Education (ESO) in Spain and in the Canary Islands. Also, we analyzed the frequencies and percentages of the test-based detected students in the sample studied.

On the other hand, to verify if there are differences in percentages in identification between Spain, the Canary Islands and in test-based detection in the sample studied (through objective test), as well as gender differences between samples, a contrast statistic was carried out using Microsoft Excel software. Specifically, classical statistical inferences based on differences in proportions have been made, as they allow comparing the proportions of two different sample distributions and formulate an inference with respect to the difference in these. In this analysis, the null hypothesis (population *p* and sample *p* are equal) is tested considering the difference in proportions as the alternative hypothesis using the following statistic (establishing the significance level of *Z* at alpha < 0.5):

$$Z=\frac{p1-p2}{\sqrt{P\left(1-P\right)\left(\frac{1}{n1}+\frac{1}{n2}\right)}}$$

where *P* is equal to

$$P=\frac{n1p1+n2p2}{n1+n2}$$

Also, Wilson Score confidence intervals for proportions have been calculated (StatsCalculators Team, 2025a), as well as effect sizes for proportion differences (StatsCalculators Team, 2025b).

## 3. Results

### 3.1. Frequencies and Percentages of Identified Students in Spain and Canary Islands, and of Test-Based Detected Students in This Study

The results, based on official data, show that the percentages of identification in the Canary Islands, and in general in Spain, increase slightly from the first to the second year, and are maintained in the third year of the ESO, although, as an exception, in the Canary Islands the percentage of girls identified decreases in the second year. In any case, the percentage of identification is between 0.54% and 1.12% in Spain (see Table 3), and between 0.77% and 1.54% in the Canary Islands (see Table 4). The differences in these results compared to those obtained in this study are imminent. On the one hand, the test-based detection percentages in this sample are between 7.30% and 11.92% (see Table 5). Another relevant aspect that differs is that the percentages decrease from the first course to the second and third. It should also be noted that in general, there are fewer girls (8.10%) detected than boys (10.35%) in our sample (see Table 5), although the difference is not as obvious as in the cases of identification of Spain (0.61% of girls and 1.07% of boys; see Table 3) and the Canary Islands (0.89% of girls and 1.44% of boys; see Table 4). 

### 3.2. Differences in Proportions Between the Identified Students in Spain and the Canary Islands, and the Test-Based Detected Students in This Study

The results show that there are significant differences between the identification ratios that occur in the whole of Spain and in the Canary Islands (higher in the Community). However, the test-based detection ratios obtained in this study show significant differences in relation to the identification proportions in Spain and in the Canary Islands, being significantly higher the test-based detection in the studied sample. These differences show small effect sizes when comparing identification proportions in Spain and the Canary Islands in all cases, but medium effect sizes (Cohen’s *h* between 0.364 and 0.475; StatsCalculators Team, 2025b) when comparing proportions of the officially identified students in the Canary Islands and the test-based detected students in our sample. Table 6 shows the differences in proportions, comparing the differences existing between Spain and the Canary Islands, and between this Autonomous Community and the test-based detection results obtained in this sample, by course and gender.

In order to determine whether the differences in the proportions of identification or test-based detection are significant by gender, differences in proportions have been calculated within each sample (Spanish, Canary, and the sample studied) in each of the courses. Results are presented in Table 7. As can be seen, the number of female participants identified in the Spanish state and the Autonomous Community of the Canary Islands is significantly lower than that detected by an objective test in our sample. However, the differences in percentages between boys and girls are not significant for the test-based detected participants of the sample in the present study.

## 4. Discussion

Considering the results of this study, the first thing that should be highlighted is that the percentages of identification of students with high abilities (HA) in the three courses of the ESO, analyzed both at Spanish level and in the Autonomous Community of the Canary Islands, are well below from the estimation of 10% of the general population made by experts (based on domain-specific definitions) in terms of the distribution of giftedness and talent in the population (Hernández & Gutiérrez, 2014). In addition, there are significant differences between the population of the Canary Islands and the rest of the Spanish State, with a higher percentage of identification in the Canary Islands than in Spain.

Nevertheless, the percentages of the test-based detection performed in the convenience sample studied are between 8.10% and 10.35%, coinciding with the percentage estimated for giftedness and talent in the population (Hernández & Gutiérrez, 2014). However, this is so among first-year students, decreasing in second- and third-year students of the ESO. It is possible that this bias is due to a higher number of repeaters, whose IQ scores could be either lower, or to a lower interest in rigorously responding to the intelligence test.

It is important to point out the relevance of detecting well the highly able students in a first step or phase, so that, once the proper evaluation or identification is performed in a second step, they can proceed to an educational intervention according to their needs and abilities. In this sense, it is worth recalling the insistent phrase of Dr. Javier Tourón, who says that the talent that does not develop is lost (Tourón, 2000). There is enough evidence to confirm that they need specific intervention programs (Pérez et al., 2017). 

Moreover, socialization and social pressures marked by gender stereotypes may revert in the behavior of adolescents with a high IQ. Adolescents could hide their high ability to please others, and for fear of rejection in their social and family environment (Muñoz, 2018). Although no evidence indicates that women are less intelligent than men, society still maintains stereotypes that question the capacity of women, as is the case of teachers (Díaz de Greñu & Anguita, 2017; Rocha et al., 2010) and parents, in addition to different gender expectations (Ayala & Mateo, 2005). If teachers and families are in charge of detecting students with possible HA, these stereotypes and gender expectations could influence their judgments and decisions and, as pointed out by Hyde (2014) and Myhill and Brackley (2004), they may hinder the detection of students with HA. This fact could be in line with official data from the Ministry of Education, Vocational Training and Sports (Ministerio de Educación, Formación Profesional y Deportes, n.d.). Whatever the subsequent reason, this study provides empirical data about the existence of significant differences in the identification of HA students in this convenience sample, to the detriment of women. However, simplifying the process by directly detecting HA students with a rigorous intelligence test with proven psychometric properties, as applied in this study, has led to disappearing the significant differences mentioned and, therefore, this could be an important step towards increasing equity and decreasing the gender gap.

Both legislation (LOE, 2006) and experts (Rodríguez-Naveiras et al., 2019) recognize the specific educational needs of this student body and the importance of early identification (Elices et al., 2006). Thus, the lack of female detection and subsequent identification is a considerable problem, as, by not being identified, they do not have access to an adequate educational response, which can result in low academic performance or even in school failure (A. M. Rodríguez, 2013; Valdés et al., 2013). Therefore, teacher training in gender issues and in high abilities is an important challenge to lead to egalitarian educational and social transformations. 

The results of this study open an opportunity to join in new procedures based on a rigorous and efficient way to detect and subsequently assess high abilities by applying an intelligence test. In all HA assessment procedures, intelligence is always measured, but often after a detection process, made with parents’ and/or teachers’ nominations (Barrera et al., 2008; Gobierno Vasco, 2019; Gobierno de Canarias, 2011; Martínez & Ollo, 2009; R. Rodríguez et al., 2017). As mentioned before, this procedure may lead to misdetection and misidentification, so the results of this study could be promising to bring a solution to this problem.

The fact of starting by comprehensively detecting the students with objective tests does not detract from the fact that, once students with higher intelligence are detected, the identification process can be optimized with other instruments that expand the assessment, especially by assessing those aspects related to personal and social adjustment and creativity, as established by the most common assessment procedures for these students. Considering the results of this study, we could say that in our sample, universal screening with a rigorous intelligence test identified a substantially larger proportion of students, including girls, than current nomination-based procedures appear to capture in administrative statistics, suggesting that such screening may reduce gender disparities in identification. Furthermore, this larger proportion would be in accordance with the 10% of giftedness and talent expected in the population by domain-specific theories of high abilities. In addition, the assessment instrument used in this study, together with the procedure applied, gives evidence that a collective intelligence test could be carried out in a group form, with a duration of around half an hour of application, and obtaining an automatic and instantaneous correction, which would simplify the work of the assessment teams involved in the detection of the HA. This could help with making the universal screening detection from an early age (Elices et al., 2006; LOE, 2006), so that the educational intervention directed to this student body could be performed without delays. Therefore, this procedure could be considered as a reliable, fast, and easy test-based detection system, so an effective gifted and talented universal screening method (Peters et al., 2024), but also a valuable procedure to increase detection and identification, to reduce gender disparities, and to facilitate equalization in participation in gifted programs in the Spanish context. Moreover, similar results have been found in studies considering universal cognitive screening procedures for the detection of highly able students (Card & Giuliano, 2016), and it would be interesting to reapply the study by using Matrices TAI or similar intelligence tests in non-convenience samples of other similar contexts and in other countries to generalize the results.

The study was not conducted without limitations. The main limitation is that the sample was selected through convenience sampling, as data collection was carried out only in schools that voluntarily agreed to participate. Therefore, it would be advisable to conduct future studies using probabilistic sampling procedures that allow for the replication of the present findings and a more accurate assessment of their generalizability. Beyond this sampling issue, additional limitations must be acknowledged. First, the detection of high ability relied exclusively on a single instrument (the Matrices-TAI), without validating the screening results against broader high-ability criteria such as creativity, academic achievement, or socio-emotional adjustment, which typically form part of comprehensive psycho-educational identification procedures. Also, a single cutoff point (IQ ≥ 120) was considered, without any sensitivity analysis across alternative thresholds. Consequently, it is not possible to estimate false positives or false negatives, nor to determine the actual educational impact that implementing a universal screening approach based solely on an intelligence test might have.

Furthermore, although detection was analyzed by gender, 19 participants selected the “other” option for the gender variable. It is important to clarify that this category did not correspond to a defined non-binary identity but rather functioned as a residual, unspecified option, which does not allow valid inferences about non-binary students. Due to the impossibility of including these cases in binary gender comparisons (male/female), they were excluded only from inferential analyses; however, their presence is acknowledged and reported descriptively in the revised version. Future research should incorporate more inclusive gender categories and examine potential differences in detection across them. Moreover, future research should also consider other potential disparities, such as socioeconomic status, immigrant background, region, or school resources, to better generalize results within a more representative sample of HA students.

## 5. Conclusions

In this study, we have concluded that a more parsimonious and evidence-based psychometric detection and assessment procedures could lead to a more efficient approach to first detect and secondly identify HA students. Also, this procedure could help increase the identification of a larger proportion of the population of highly able students and decrease the gender gap, thus contributing to the end of gender inequalities in the detection and identification of high abilities. This could help to improve the opportunities given for these students to have access to an adequate educational response that allows the correct development of their potential.

## Figures and Tables

**Table 1:** Frequencies and percentages of students identified with HA in relation to the total of non-university students in Spain and in the Autonomous Communities.

Autonomous Community	Total Number of Students		Identified Students			
	Male	Female	Male		Female	
Spain	4,246,506	3,985,789	26,601	0.63%	14,315	0.36%
Region of Murcia	158,401	141,529	1937	1.22%	1110	0.78%
Principality of Asturias	70,333	65,708	975	1.39%	462	0.70%
Andalucia	821,694	776,806	10,356	1.26%	6639	0.86%
Balearic Islands	100,454	92,467	1223	1.22%	587	0.64%
La Rioja	28,765	26,926	321	1.12%	147	0.55%
Canaries	173,548	167,028	1516	0.87%	877	0.53%
Galicia	208,118	193,450	1613	0.78%	700	0.36%
Navarra	60,560	56,167	468	0.77%	205	0.37%
Castile and Leon	177,368	164,387	850	0.48%	252	0.15%
Extremadura	88,608	83,808	366	0.41%	144	0.17%
Madrid	629,481	593,469	2381	0.38%	1103	0.19%
Castile-La Mancha	186,844	175,059	589	0.32%	224	0.13%
Catalonia	706,862	676,124	2166	0.31%	1124	0.17%
Basque Country	196,451	176,015	650	0.33%	287	0.16%
Cantabria	48,346	45,748	194	0.40%	70	0.15%
C. Valencia	455,100	425,913	840	0.19%	312	0.07%
Ceuta	10,461	9893	11	0.11%	20	0.20%
Aragon	114,224	105,045	132	0.12%	48	0.05%
Melilla	10,852	10,247	13	0.12%	4	0.04%

Source: own development, based on data from the Ministry of Education, Vocational Training and Sports (Ministerio de Educación, Formación Profesional y Deportes, n.d.) Course 2020–2021.

**Table 2:** Participants based on self-defined gender.

	Male	Female	Other	Total
1st ESO	193	209	7	409
2nd ESO	219	233	5	457
3rd ESO	187	175	7	369
Total	599	617	19	1235

**Table 3:** Identification of students with HA with respect to the total of students from first to third years of the Compulsory Second Education (ESO) in Spain by gender.

		Both Genders	Male	Female
1st ESO	Total	536,902	279,279	257,623
	HA	4129	2741	1388
	%	0.77	0.98	0.54
2nd ESO	Total	537,104	280,213	256,891
	HA	4778	3132	1646
	%	0.89	1.12	0.64
3rd ESO	Total	499,701	254,841	244,860
	HA	4420	2804	1616
	%	0.88	1.1	0.66
Total ESO	Total	1,573,707	814,333	759,374
	HA	13,327	8677	4650
	%	0.85	1.07	0.61

Source: own development, based on data from the Ministry of Education, Vocational Training and Sports (Ministerio de Educación, Formación Profesional y Deportes, n.d.). Course 2020–2021.

**Table 4:** Identification of students with HA with relation to the total of students from first to third of the Compulsory Second Education (ESO) in the Canary Islands by gender.

		Both Genders	Male	Female
1st ESO	Total	22,276	11,613	10,663
	HA	248	153	95
	%	1.11	1.32	0.89
2nd ESO	Total	23,454	12,331	11,123
	HA	266	180	86
	%	1.13	1.46	0.77
3rd ESO	Total	22,392	11,308	11,084
	HA	286	174	112
	%	1.28	1.54	1.01
Total ESO	Total	68,122	35,252	32,870
	HA	800	507	293
	%	1.17	1.44	0.89

Source: own development, based on data from the Ministry of Education, Vocational Training and Sports (Ministerio de Educación, Formación Profesional y Deportes, n.d.). Course 2020–2021.

**Table 5:** Test-based detection of students with HA in relation to the total of students of this sample, from first to third year of the Compulsory Second Education (ESO) by gender.

		Both Genders	Male	Female
1st ESO	Total	402	193	209
	HA	42	23	19
	%	10.45	11.92	9.09
2nd ESO	Total	452	219	233
	HA	39	22	17
	%	8.63	10.05	7.3
3rd ESO	Total	362	187	175
	HA	31	17	14
	%	8.56	9.09	8
Total ESO	Total	1216	599	617
	HA	112	62	50
	%	9.21	10.35	8.1

**Table 6:** Differences in identification percentages between Spain, the Canary Islands and the test-based detection percentages of the sample studied by gender.

Course		Both Genders				Male				Female			
		P	95% C.I.	Z	Cohen’s h	P	95% C.I.	Z	Cohen’s h	P	95% C.I.	Z	Cohen’s h
1st ESO	Spain	0.0077	[0.0075, 0.0079]	−5.7132	0.036	0.0098	[0.0095, 0.0102]	−3.5752	0.032	0.0054	[0.0051, 0.0057]	−4.8062	0.042
	Canaries	0.011	[0.0098, 0.0126]			0.0132	[0.0113, 0.0154]			0.0089	[0.0073, 0.0109]		
	Canaries	0.011	[0.0098, 0.0126]	−16.3790	0.447	0.0132	[0.0113, 0.0154]	−12.0516	0.475	0.0089	[0.0073, 0.0109]	−11.5256	0.423
	Our Sample	0.1045	[0.0782, 0.1382]			0.1192	[0.0807, 0.1725]			0.0909	[0.059, 0.1376]		
2nd ESO	Spain	0.0089	[0.0086, 0.0092]	−3.8822	0.024	0.0112	[0.0108, 0.0116]	−10.0082	0.03	0.0064	[0.0061, 0.0067]	−1.7065	0.016
	Canaries	0.0113	[0.0101, 0.0128]			0.0146	[0.0126, 0.0169]			0.0077	[0.0063, 0.0095]		
	Canaries	0.0113	[0.0101, 0.0128]	−14.0618	0.383	0.0146	[0.0126, 0.0169]	−8.0132	0.403	0.0077	[0.0063, 0.0095]	−10.3943	0.371
	Our Sample	0.0863	[0.0638, 0.1158]			0.1005	[0.0673, 0.1474]			0.073	[0.0673, 0.1474]		
3rd ESO	Spain	0.0088	[0.0086, 0.0091]	−6.0830	0.038	0.011	[0.0106, 0.0114]	−4.3373	0.039	0.0066	[0.0063, 0.0069]	−4.4075	0.039
	Canaries	0.0128	[0.0114, 0.0143]			0.0154	[0.0133, 0.0178]			0.0101	[0.0084, 0.0121]		
	Canaries	0.0128	[0.0114, 0.0143]	−12.0481	0.367	0.0154	[0.0133, 0.0178]	−8.0740	0.364	0.0101	[0.0084, 0.0121]	−8.7212	0.372
	Our Sample	0.0872	[0.061, 0.119]			0.0909	[0.0575, 0.1408]			0.08	[0.0483, 0.1298]		

Note: P = proportion above IQ ≥ 120; Z = z-statistics for differences in proportions.

**Table 7:** Differences in gender identification or test-based detection percentages in each sample.

Course	Gender	Spain				Canaries				Studied Sample			
		P	95% C.I.	Z	Cohen’s h	P	95% C.I.	Z	Cohen’s h	P	95% C.I.	Z	Cohen’s h
1st ESO	Male	0.0098	[0.0095, 0.0102]	18.55	0.052	0.0132	[0.0113, 0.0154]	3.01	0.041	0.1192	[0.0807, 0.1725]	0.93	0.092
	Female	0.0054	[0.0051, 0.0057]			0.0089	[0.0073, 0.0109]			0.0909	[0.059, 0.1376]		
2nd ESO	Male	0.0112	[0.0108, 0.0116]	18.596	0.052	0.0146	[0.0126, 0.0169]	4.958	0.066	0.1005	[0.0673, 0.1474]	1.04	0.098
	Female	0.0064	[0.0061, 0.0067]			0.0077	[0.0063, 0.0095]			0.073	[0.0673, 0.1474]		
3rd ESO	Male	0.011	[0.0106, 0.0114]	16.618	0.048	0.0154	[0.0133, 0.0178]	3.519	0.047	0.0909	[0.0575, 0.1408]	0.37	0.039
	Female	0.0066	[0.0063, 0.0069]			0.0101	[0.0084, 0.0121]			0.08	[0.0483, 0.1298]		

Note: P = proportion above IQ ≥ 120; Z = z-statistics for differences in proportions.

## Data Availability

Data will be available on request by contacting the corresponding author.
